# Surveying End-of-Life Medical Decisions in France: Evaluation of an Innovative Mixed-Mode Data Collection Strategy

**DOI:** 10.2196/ijmr.3712

**Published:** 2016-02-18

**Authors:** Stephane Legleye, Sophie Pennec, Alain Monnier, Amandine Stephan, Nicolas Brouard, Johan Bilsen, Joachim Cohen

**Affiliations:** ^1^ Institut National d'études Démographiques Department of Survey and Sampling Paris France; ^2^ Institut National d'études Démographiques Paris France; ^3^ Vrije Universiteit Brussel Head Department of Public Health Brussels Belgium; ^4^ Vrije Universiteit Brussel End-of-Life Group Brussels Belgium

**Keywords:** end-of-life decisions, France, methodology, mixed-mode survey

## Abstract

**Background:**

Monitoring medical decisions at the end of life has become an important issue in many societies. Built on previous European experiences, the survey and project Fin de Vie en France (“End of Life in France,” or EOLF) was conducted in 2010 to provide an overview of medical end-of-life decisions in France.

**Objective:**

To describe the methodology of EOLF and evaluate the effects of design innovations on data quality.

**Methods:**

EOLF used a mixed-mode data collection strategy (paper and Internet) along with follow-up campaigns that employed various contact modes (paper and telephone), all of which were gathered from various institutions (research team, hospital, and medical authorities at the regional level). A telephone nonresponse survey was also used. Through descriptive statistics and multivariate logistic regressions, these innovations were assessed in terms of their effects on the response rate, quality of the sample, and differences between Web-based and paper questionnaires.

**Results:**

The participation rate was 40.0% (n=5217). The respondent sample was very close to the sampling frame. The Web-based questionnaires represented only 26.8% of the questionnaires, and the Web-based secured procedure led to limitations in data management. The follow-up campaigns had a strong effect on participation, especially for paper questionnaires. With higher participation rates (63.21% and 63.74%), the telephone follow-up and nonresponse surveys showed that only a very low proportion of physicians refused to participate because of the topic or the absence of financial incentive. A multivariate analysis showed that physicians who answered on the Internet reported less medication to hasten death, and that they more often took no medical decisions in the end-of-life process.

**Conclusions:**

Varying contact modes is a useful strategy. Using a mixed-mode design is interesting, but selection and measurement effects must be studied further in this sensitive field.

## Introduction

Improved living conditions, public health initiatives, and advances in medical care in most developed countries have contributed to a rise in life expectancies and a significant shift in the causes of death. Deaths from acute infectious diseases have declined, whereas deaths from chronic and degenerative diseases such as cancer and cardiovascular pathologies have increased [1,2]. More and more, people are dying at older ages, often in hospitals where they are permanently assisted by physicians and other health care workers who are directly involved in the dying process. These professionals can administer drugs to alleviate pain or other symptoms, and withhold or withdraw treatment to prolong life; however, the deliberate hastening of death is legally forbidden in France. Consequently, the quality of medical care at the end of life has become a significant concern in many societies, and it has become necessary to monitor related medical decisions reliably, to provide data to inform debates on this sensitive subject. Legislation concerning medical end-of-life decisions varies widely in Europe [3]. Over the last few decades, several European countries have performed single surveys or a series of surveys on this topic, including the Netherlands, Belgium, Denmark, Sweden, Switzerland, and Italy [4-8]. In France, major changes were introduced to the legislation in 2005 [9]. However, no nationwide representative survey has ever been undertaken, and the only available data has come from studies in hospitals and emergency wards [10-13]. Thus, there is a lack of knowledge of overall medical practices.

One of the main obstacles to conducting population-based studies is that health care professionals are recognized as a “hard-to-reach” population [14], resulting in high nonresponse rates. In combination with substantial differences between nonrespondents and respondents, this can lead to considerable nonresponse bias [15], which undermines the validity of these studies. For this reason, efforts aimed at improving participation rates must be concerned with every aspect of a protocol [16-18]. First, positive impacts have been noted using the following methods: varying contact modes and/or the form of postal contact (letters, cards, telephone, etc) [17], personalized cover letters, replacement questionnaires combined with a high number of follow-up contacts, and a long data collection period [19,20]. Second, mixing data collection modes (eg, mail and internet) may show positive results, although postal questionnaires are often favored over telephone and Web-based surveys [21,22]. Finally, surveying a sample of nonrespondents may also improve data quality, through the assessment of reasons for refusal and/or the determination of sociodemographic or professional characteristics of nonrespondents [17,23]. This latter technique was used in the 2001 end-of-life decision surveys in the Netherlands, Switzerland, and Sweden [24], as well as in the 2007 Belgium survey [25].

The survey *Fin de Vie en France* (“End of Life in France,” abbreviated as EOLF) was conducted in 2010 by the Institut national d’études démographiques (INED), with the purpose of describing end-of-life medical decisions in the French context [26]. Compared to previous surveys, EOLF comprised several innovations, including a mixed-mode procedure (internet and postal questionnaire) as well as postal and telephone follow-ups, combined with postal or email reminders sent by the medical authorities (hospitals and regional health agencies). It also comprised a nonrespondent telephone survey to assess nonresponse bias.

The aim of this paper was to describe and evaluate the methodological innovations of EOLF and to assess their impact on data collection quality. It describes response rates, representativeness of the sample, motives for nonresponse, and differences resulting from the data collection modes. Regarding the mixed-mode methodology, we assessed whether the choice of Internet over paper questionnaires was linked to the characteristics of the participating physician or of the deceased person, and whether this choice had an impact on the reporting of end-of-life medical decisions.

## Methods

### Retrospective Design

We chose to sample deaths and not physicians [4-8] for the same reasons given by Chambaere et al [25]. A representative sample of 14,999 deaths was selected by the CepiDc (*Centre d'épidémiologie sur les causes médicales de décès*) de l’Institut National de la Santé et de la Recherche Médicale (INSERM), and was drawn using systematic random sampling (sorted by age, gender, place of death, and region of residence of the deceased person [27]) from among 47,872 deaths of persons aged 18 and over that occurred in continental France in December 2009.

The certifying physician was identified, and a questionnaire about the selected deceased person(s) was mailed to her/him with instructions for replying. Physicians could respond either by post or by using a specially developed secure Web-based questionnaire. When physicians had more than one death in the sample, for each death, we provided an identifier and password to use in the Web-based mode and a questionnaire with a prepaid envelope to reply by post.

### Anonymity and Follow-Up

While the Belgian survey procedure employed a lawyer as a third party, preliminary discussions in France concluded that any mention of a lawyer for this kind of survey would provoke reluctance rather than reassure physicians to participate. We used the services of a specialized hospital department to play the role of the trusted third party for the paper administration [28]; the members of this service also entered the paper-based questionnaire responses manually using the secure Web-based questionnaire that was developed.

The Web-based administration adapted the same approach by involving a trusted virtual party [29]. This method used more than one Web server; answers entered by the practitioner were neither sent to nor temporarily saved on the first Web server (the one to which they logged on). Instead, the responses were sent to a second server using .xml files, for which filenames included a second and different irreversible hash chain of the death identity. In addition, access to log files was not activated on this host Web server, in order to suppress information regarding Internet protocol addresses. Furthermore, no electronic acknowledgment was sent to physicians, and all .xml files received had the same date of creation on the host server. This overprotection of the practitioner’s identity contrasts with current philosophy of Web security, which consists of tracking connections to a server and forcing browser clients to dialog with a unique, well-identified server. The material sent to physicians explained how their anonymity was ensured.

No link could be established between the physician and the information in the death certificate (see *Questionnaires* and *Data Quality Check*).

### Materials

#### Contact Material

Following the recommendations of Dillman [17], the first mailing was personalized (name and address of the physician), and the leading scientific institutions were clearly identified on the envelope. It contained: an invitation letter, which was signed by the heads of the leading scientific institutions involved and which also explained the importance of the study; a follow-up card for cases in which the physician who had only certified the death could fill in the name and address of the physician who had actually treated the patient until her/his death; a color-printed questionnaire; and a prepaid envelope for returning the questionnaire or card. A leaflet was also provided, which presented the survey and a flyer describing how the anonymity of respondents was ensured. For anonymity purposes, the completed questionnaire was placed in a sealed envelope inside the prepaid envelope (as for absentee postal voting).

The survey was also advertised by the main French medical bodies/authorities and by regional health agencies before and during data collection, as well as in a press release issued just before the survey went out to the field.

#### Questionnaires

The questionnaire was 20 pages long in A5 booklet form, with only closed questions and no space for writing any comments, as required by the French data protection committee (CNIL). The questionnaire of around 100 questions comprised the following sections: characteristics of the responding physician; characteristics of the deceased person; place of death; treatments (palliative care, sedation, etc); information related to the 2005 law (health-care proxy/surrogate, living will or advanced medical directives, discussion of last medical decision with patient or surrogate, and discussion with colleagues/collegiality of discussion); medical end-of-life decisions; wish of the deceased person to hasten death and/or euthanasia; medical practitioners or nursing staff involved in the end-of-life period; and visits of family/friends [30].

Although the age and sex of the deceased person, region of death, and month of death were identified on the death certificate and sent to the physician to identify the death, we asked for some of this information in the questionnaire because, in order to preserve anonymity, questionnaires were not merged with death certificates.

The questionnaires used in the phone-call campaign and in the nonresponse survey (which was also administered by phone) were shorter than the main questionnaire. Although these questionnaires focused on the motives for nonresponse, they also included several questions identical to those in the main questionnaire, for describing the physicians’ characteristics.

### Data Collection

#### Identification of the Certifying Physician

Names of physicians and their professional addresses were identified by their signatures and stamps on the death certificates and entered manually. In ambiguous or unreadable cases, names and addresses were requested from the mayor’s office that recorded the certificates. The names and addresses of each physician were checked manually using the Internet and the administrative register of all physicians in France. The result of this preliminary phase is presented in Table 1.

**Table 1:** Identification of physicians/death certificates.^a^

Sample	n or %
Initial sample of death certificates (1)	14,999
Death certificates with directly identified physician (2)	11,412
Death certificates with unidentified physician, sent to mayor’s offices (3)	3587
Physician identified by the mayor’s offices (4)	2828
Impossible to identify the physician (5.1)	759
Certificate disregarded due to the cut-off of 4 certificates per physician, except in cases of heads of department (5.2)	160
Final sample, death certificates (6)=(2)+(4)−(5.2)	14,080
Number of physicians	11,828
Proportion of unused death certificates (10)=[(5.1)+(5.2)]/(1)	6.12
Proportion of used death certificates (11)=(6)/(1)	93.87

^a^The figures in parentheses are useful to compute % in this table.

#### Eligibility of the Physician

The major reasons for not including a physician in the study were the inability to find a professional workplace address or a late reply from the mayor’s office (a cut-off point was set for the end of May, and we discarded late returns from the Mayor’s office). To reduce refusal due to workload, we set a limit of four questionnaires per physician. An exception was made for department heads in institutions, because we discovered during the pilot survey that some heads of departments signed the certificate for most, if not all, deaths that occurred in their department (often treated by another physician). Thus, we anticipated that department heads would distribute questionnaires to corresponding treating physicians.

#### Fieldwork Stages

Data collection comprised three stages. The first stage was a postal phase. The initial mailing, including the questionnaire, was sent on 2010 May 25. A first postal reminder letter was sent to the nonrespondents two weeks later. Two weeks after that reminder, a third mailing was sent with the same material as the first one, but with adapted text. We avoided sending letters during the summer holiday period. A fourth and last reminder was sent to nonrespondents in September. The entire procedure was enhanced in three ways. First, there was a press release issued just before the survey. Second, we sent a letter in July to all directors of hospitals with at least one physician who had been selected for our sample, to ask him/her to convince their physicians to participate. Third, regional health agencies sent a similar motivational letter to institutions during the second half of August, asking them to communicate with physicians about the study and requesting that they pay attention to it and participate if they had been selected.

The second stage of data collection began on 17 Jun 2010 with a telephone campaign. At that time, there were 10,677 nonrespondent physicians, and a telephone number was known for 10,582 of them. The aim was to personally contact 7,000 of these physicians by phone to convince them to participate or, if they declared that they did not want to participate in the survey, to report their motives for refusal (for financial reasons, we could not contact all physicians). For this purpose, a sample of 9210 nonrespondents was drawn from among the 10,677 nonrespondents at the beginning of the telephone campaign (86.26%). This sample comprised all physicians who had signed at least two death certificates (n=1565), and others who were included randomly. Each number was to be contacted up to 16 times before being abandoned (globally, the mean number of contact attempts was 4.8). This number of call attempts was chosen based on efficiency and budgetary considerations. In parallel, a third letter, similar to the second one, was sent to 95 nonrespondent physicians whose phone number was unknown. This stage took place in two phases: 17 June-21 July, and 4 October-5 November.

**Figure 1:**
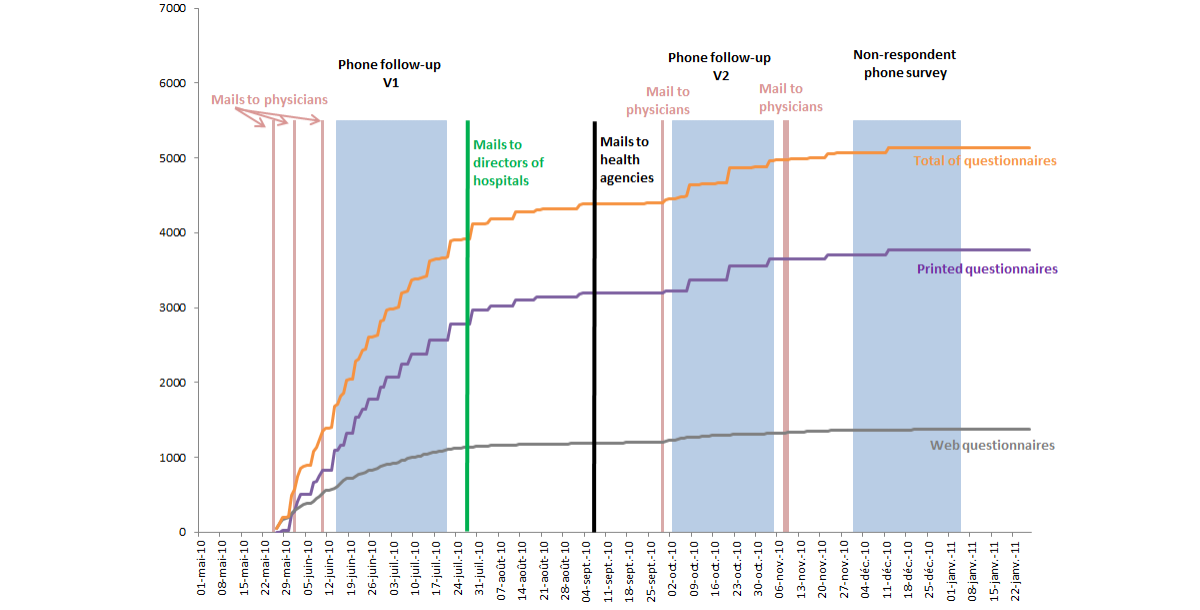
Schedule of data collection fieldwork and number of questionnaires by data collection mode.

The third stage was a telephone survey of 1080 final nonrespondent physicians. It began on 7 December 2010 and ended on 7 January 2011. Each phone number was to be contacted up to 20 times before being abandoned. The goal of this survey was only to collect the motives for nonresponse, along with basic characteristics of the physicians (age group, sex, and specialty). To obtain an accurate measure of these, we set a high number of call attempts because we anticipated that these physicians would be very hard to contact (Figure 1 shows the data collection modes).

During all fieldwork, a hotline (8 am-8 pm) was offered to provide information about the survey and to resend materials to physicians in case of loss.

### Data Quality Check

We checked that the month of death reported by physicians in the questionnaire was December 2009, to be sure that the deaths they were reporting on were those selected in the sample and not arbitrary ones (such as the most recent or a more interesting case). Previous end-of-life decision surveys in other countries did not take this precaution.

### Weighting

The final respondent sample was weighted using a calibration procedure [31], considering age × sex, and region and place of death, as observed in the initial sample of deaths.

### Ethical Considerations

This survey was approved by the *Comité Consultatif sur le Traitement de l’Information en matière de Recherche dans le domaine de la Santé* (CCTIRS) in January 2010 and authorized by CNIL (authorization number 1410166).

### Statistical Analysis

Samples were described using percentages and bivariate analyses with Pearson chi-square tests. Three multivariate logistic models (providing adjusted odds ratios and 95% confidence intervals) were also computed when comparing Web-based and paper questionnaires. These models tested whether the choice of Web-based questionnaires was linked to physician characteristics (Model 1), death characteristics (Model 2), or both sets of characteristics (Model 3). All statistics were computed using SAS V9.3 and were nonweighted unless specified.

## Results

### Preliminary Identification of Death Certificates, Physicians, and Participation Rate

Overall, 14,080 death certificates (93.87% of the initial sample of deaths) with identified physicians were available for the survey, corresponding to 11,828 different physicians (Table 1). The final sample was reduced to 13,460 deaths because of postal address problems (changes in professional location, etc). From this sample, 5217 questionnaires were completed and returned. This led to a participation rate of 40.02% [32].

We used the standard Response Rate 2 from the American Association for Public Opinion Research (AAPOR). The formula used was 5217 questionnaires/(5217 questionnaires + 1506 refusals + 449 letters not delivered + 561 physicians who could not respond because the survey did not concern them [sudden death, not the physician in charge of the patient, could not remember the case or could not find the file] + 49 other reasons for nonresponse [eg, death of respondent, retirement, not available during data collection] + E[6287 neither responding nor refusing]) = 40.02%. E is the estimation of the proportion of eligible cases (in this case, 92.10%). E was determined by the ratio of the sum of questionnaires + refusals + others, to the sum of questionnaires + refusals + others + non-eligible persons. We counted as non-eligible those who mentioned in the follow-up survey that they did not respond because the survey did not concern them (not in charge of the patient, not a forensic scientist, could not remember or find the file of the person, etc).

An additional analysis of the follow-up file (not mentioned in the table) showed that the response rate varied with the patients’ characteristics. It varied by age of the deceased (from 42.12% for deceased individuals aged 18-39 to 35.03% for those aged 90+), place of death (from 29.15% for nursing homes to 17.17% for public places and 40.20% for public hospitals), and region of residence (from 30.76% in the Mediterranean region to 40.42% in the East). It also varied with the physicians’ characteristics: participation of women was higher (43.25% vs 34.14%), and type of practice exhibited variation (from 30.92% for those working in private practice—regardless of whether they were general practitioners or specialists—to 35.67% for those in emergency services and 39.12% for those working in other services at a public hospital).

### Data Quality Check

During the data quality check, 311 questionnaires were discarded because either the reported month of death was not December (230) or the month of death was missing (81). Chi-square tests showed that these 311 questionnaires were slightly different for some medical decisions (treatment prolonging life: 35.37% vs 31.92%, *P*=.26; withdrawal from treatment: 16.10% vs 21.81%, *P*=.0041; treatment to alleviate pain: 44.37% vs 40.85%, *P*=.221; and medication to hasten death: 2.25% vs 0.74%, *P*=.004). Another 15 questionnaires could not be used because of computer problems encountered by the physicians. We thus decided to exclude all these deaths from the analysis: the final sample contained 4891 valid questionnaires.

### Sample Structure

Table 2 exhibits the nonweighted sociodemographic structure of the initial sample drawn from the national death register and of the final death sample that was analyzed. This structure was close to the actual structure of all deaths in December, except for a small overrepresentation of deaths in public hospitals.

### Telephone Campaign

The second stage of data collection was a telephone campaign: 6169 physicians/secretariats were contacted from the sample of 9220 nonrespondents at that time. Finally, 5421 physicians were spoken to personally and participated in the telephone campaign (Table 3).

Among these 5421, 505 (9.32%) felt the EOLF survey did not concern them (and thus the telephone campaign), 1106 (20.38%) reported that they were currently participating in the main survey, 2621 (48.35%) stated that they wanted to participate in the EOLF survey, while 1189 (21.93%) explicitly refused to participate. Of the physicians who wanted to participate, 1098 (41.89%) were not aware of the survey, 542 (20.67%) asked for the material to be sent again, and 981 (37.43%) promised to participate soon. The response rate to the telephone campaign was thus 63.21% (following the AAPOR standard Response Rate 2). Among the 1189 refusing physicians, 332 (27.92%) indicated that they never participate in surveys, 603 (50.71%) said that they were currently too busy, and 36 (3.03%) mentioned the absence of a financial incentive. In addition, 100 (8.41%) mentioned reasons related to the usefulness of such a survey, 29 (2.44%) reported that the subject was too personal and/or too sensitive, and another 25 (2.10%) reported other reasons related to the methodology of the survey. The other 20 refusing physicians did not specify their reasons (1.68%). Overall, the telephone campaign seemed to strongly improve the participation rate, as shown in Figure 1. The effects of the other mailings and follow-up interventions of the hospitals and health agencies were less clear, although they may also have contributed to the success of the phone campaign. The effects of the telephone campaigns on the Web-based questionnaires appeared modest compared to those on the paper questionnaires: the number of collected Web-based questionnaires was almost stable after the first phone follow-up.

**Table 2:** Sociodemographic characteristics of the sample of deaths: initial sample, selected sample, and returned questionnaires (%).

		Initial sample N=14,999		Selected sample (after removals) N=14,080		Final sample/analysis file^a^ (nonweighted) N=4891		All deaths in December^b^ N=47,986	
		n	%	n	%	n	%	n	%
**Sex**									
	Men	7598	50.66	7115	50.53	2454	50.17	24,370	50.79
	Women	7401	49.34	6965	49.47	2437	49.83	23,616	49.21
**Age**									
	18-49 years	775	5.17	712	5.06	290	5.88	2585	5.39
	50-69 years	2790	18.60	1105	18.34	946	19.34	9106	18.98
	70-79 years	3007	20.05	2835	20.13	980	20.04	9478	19.75
	80-89 years	5785	38.57	5439	38.63	1839	37.60	18,443	38.43
	≥90 years	2642	17.61	2512	17.84	836	17.09	8374	17.45
**Place of patient death**									
	Home	3426	22.84	3261	23.16	1013	20.71	12,797	26.67
	Public hospital	7415	49.44	6965	49.47	2705	55.31	23,460	48.89
	Private hospital	1334	8.89	1229	8.73	348	7.12	4015	8.37
	Nursing home	2310	15.40	2237	15.89	673	13.76	5625	11.72
	Public place	174	1.16	159	1.13	82	1.68	643	1.34
	Other	238	1.59	229	1.63	70	1.43	1446	3.01
	Missing	102	0.68		0.0		0.0		0.0
**Region of death**									
	Ile de France	2005	13.37	1847	13.12	629	12.85	6434	13.41
	Bassin Parisien	2679	17.86	2535	18.00	919	18.79	8777	18.28
	Nord	1022	6.81	935	6.64	326	6.67	3204	6.68
	Est	1337	8.91	1274	9.05	500	10.23	4178	8.70
	Ouest	2273	15.15	2144	15.23	767	15.68	7132	14.87
	Sud-Ouest	1900	11.75	1786	12.68	561	11.48	5977	12.46
	Centre-Est	1763	11.75	1657	11.77	633	12.93	5547	11.56
	Méditerranée	2020	13.47	1902	13.51	555	11.36	6737	14.04

^a^As reported on the questionnaire by the physician.

^b^Based on all coded death certificate data from December.

**Table 3:** Results of the telephone campaign and motives for nonresponse.^a^

Results			N	%
**Contact achieved**			6169	66.91
	Physician personally contacted and participated in the telephone campaign		5421	58.80
	Phone call blocked by the secretariat		296	3.21
	Contact made but physician not reachable		452	4.90
No contact achieved			3051	33.09
**Personally contacted physicians who participated in the telephone campaign**			5421	100.00
	Explicitly refused to participate in the main survey		1189	21.93
	Did not concern them^b^		505	9.32
	**Participation in the main survey was in progress**		1106	20.38
		Already sent the questionnaire	506	9.33
		Transferred the material to the right physician	600	11.07
	**Wanted to participate in the main survey**		2621	48.35
		Was not aware of the survey	1098	20.25
		Asked for the material to be sent again	542	10.00
		Promised to participate soon	981	18.10
**Motives for explicit refusals to participate in the main survey (multiple responses)**			1189	100.00
	Lack of time		603	50.71
	Never participate in surveys		332	27.92
	Too many surveys		90	7.57
	Absence of financial incentive		36	3.03
	Questionnaire too long		49	4.12
	Reason related to survey usefulness		100	8.41
	Reason related to survey topic		29	2.44
	Reason related to survey methodology		25	2.10
	Not specified		20	1.68

^a^Initial nonresponse sample (N) = 9220.

^b^Forensic scientists are the certifying physician in cases of violent or suspicious deaths.

### Survey of Nonrespondents

The survey of the final nonrespondents, conducted by phone from December 2010 to January 2011 (the third stage of data collection), used a random sample of 1080 physicians with valid phone numbers who did not express explicit refusals but also did not respond during fieldwork. Contact was made in 957 cases, and 684 physicians could be contacted personally (Table 4). Finally, 547 physicians agreed to participate in the nonrespondent survey (79.97% of all personally contacted physicians); 38 reported that they had already participated in the main survey and 52 said that they would still participate, leading to a response rate of 63.74% (as above, this rate is computed following the AAPOR standard Response Rate 2).

Among respondents, the most frequent motive for nonresponse to the main survey was lack of time (53.02%). However, some survey-specific motives were frequently reported: it was impossible to remember the deceased person, or it was too difficult to find the medical file (14.26% and 9.69%). Almost 6% (5.85%) of the physicians reported having been unaware of the survey, indicating difficulties in contacting them personally.

**Table 4:** Results of the telephone nonrespondent survey (third stage) and motives for nonresponse (N=1080).

Result			N	%
**No answer obtained**			123	11.39
	Always get the answering machine		18	1.67
	No answer after 20 call-backs		38	3.52
	Number not valid		46	4.26
	Other reasons		21	1.94
**Answer obtained**			957	88.61
	**Physician personally contacted**		684	63.33
		Participation in the nonrespondent survey^a^	547	50.65
		Refusal to participate in the nonrespondent survey	47	4.35
		Already answered the main survey	38	3.52
		Not aware, will participate in the main survey	52	4.81
	Refusal from secretariat		108	10.0
	Refusal from the physician		36	3.33
	Impossible, not eligible		129	11.94
**Motives for nonresponse (multiple responses)** ^b^			547	
	Lack of time		290	53.02
	Does not remember the case		78	14.26
	Was not in charge of the deceased person		64	11.7
	Forgot to answer/lost the questionnaire		53	9.69
	Too difficult to find the medical file		53	9.69
	Unaware of the survey		32	5.85
	Questionnaire too long		28	5.12
	Reason related to the survey topic		6	1.10
	Reason related to the survey methodology		7	1.28

^a^A total of 78 physicians who answered the nonresponse survey mentioned that they had already answered the main survey or that they would do so. ^b^The percentages do not add to 100 because this was a multiple-response question.

### Differences Between Internet and Paper Responses

Overall, 73.21% of the questionnaires (n=3557) were on paper while 26.78% (n=1334) were collected through the secure Web-based questionnaire. As shown in Table 5, physicians who chose the Internet were more often male, younger, and working in large towns or institutions. Compared to general practitioners (GPs) in private practice, almost all specialists were more likely to choose the Internet, especially anesthesiologists and GPs in hospitals (but not oncologists or cardiologists). Causes of death were not exactly similar in both modes (*P*=.033): cancer was more frequent for the paper questionnaire (28.45% vs 25.19%, *P*=.019) and infectious diseases more frequent for the Web-based questionnaire (8.80% vs 6.28%, *P*=.003). Medical decisions were similarly distributed in the two samples (*P*=.114), although medications to hasten death were more frequent for the paper questionnaire (0.96% vs 0.33%, *P*=.036, crude odds ratio [OR] 0.35, 95% CI 0.13-0.96).

To test whether these bivariate differences could be explained by patient or physician characteristics, we ran three logistic models. Controlling for physician characteristics, Model 1 shows that physician differences between the Web-based and paper questionnaires remained, and that reporting “no decision” was more frequent for Web-based questionnaires (OR 1.44, 95% CI 1.08-1.71).

**Table 5:** Comparison of questionnaires completed on the Internet versus on paper: percentages, adjusted odds ratios (OR), and 95% confidence intervals (95% CI).

Characteristics		Internet sample n^a^=1334	Paper sample n^a^=3557		Physician characteristic (Model 1)		Patients characteristic (Model 2)		Physician and patient characteristics (Model 3)	
		%	%	*P* ^b^	OR	95% CI	OR	95% CI	OR	95% CI
**Physician gender**				.001						
	Men	73.45	65.27		1		1			
	Women^c^	26.55	34.73		0.61	0.52-0.72			0.60	0.51-0.70
**Physician age**				.001						
	18-39 years old	29.20	21.40		1				1	
	40-49	32.16	28.23		0.87	0.72-1.04			0.86	0.72-1.04
	50-59	27.96	35.81		0.56	0.46-0.68			0.55	0.45-0.67
	60+	10.68	14.56		0.54	0.42-0.70			0.52	0.40-0.68
**Physician medical specialty**				.001						
	GPs in private practice	11.21	20.69		1				1	
	Oncologists	3.24	4.00		1.05	0.68-1.63			1.02	0.62-1.67
	Cardiologists	2.89	3.04		1.16	0.73-1.84			1.15	0.69-1.91
	Geriatrists in hospitals	6.57	7.56		1.54	1.10-2.16			1.51	1.04-2.17
	Geriatrists elsewhere	10.92	11.91		1.44	1.05-1.96			1.42	0.99-2.04
	Emergency physicians	17.78	13.54		1.55	1.14-2.11			1.41	1.02-1.95
	Anesthesiologists	18.72	9.25		2.39	1.76-3.25			2.18	1.50-3.16
	Other specialist in hospital	3.74	3.80		1.29	0.86-1.94			1.24	0.79-1.94
	Other specialist outside hospital	11.87	12.50		1.20	0.89-1.64			1.18	0.82-1.71
	Other GPs	7.36	8.32		1.46	1.07-2.01			1.44	1.03-2.02
	GPs in hospitals	5.71	5.40		1.75	1.21-2.54			1.69	1.12-2.57
**Physician town size**				.001						
	>200,000	25.59	15.26		1				1	
	<10,000	17.90	30.19		0.58	0.45-0.75			0.59	0.45-0.76
	10,000-20,000	11.02	12.44		0.84	0.66-1.07			0.81	0.63-1.05
	20,000-100,000	32.66	32.64		0.62	0.51-0.75			0.61	0.50-0.74
	100,000-200,000	12.83	9.47		0.50	0.39-0.63			0.49	0.39-0.63
**Death certificates (3 months)**				.001						
	0	3.42	6.15		1				1	
	1-2	18.40	22.01		1.26	0.87-1.83			1.28	0.88-1.85
	3-4	27.96	28.53		1.29	0.87-1.92			1.34	0.90-2.00
	5-9	27.91	25.66		1.27	0.81-1.98			1.27	0.81-2.01
	10-19	15.73	12.60		1.23	0.85-1.78			1.27	0.88-1.85
	20+	6.57	5.06		1.19	0.81-1.73			1.20	0.82-1.76
**Physician medical decision**				.0242							
	Sudden death	17.53	16.68		1		1		1	
	Life-prolonging treatment	12.62	12.04		1.05	0.80-1.36	0.90	0.69-1.16	1.02	0.77-1.35
	Treatment withheld	13.12	15.10		1.02	0.79-1.32	0.88	0.68-1.14	1.05	0.80-1.39
	Treatment withdrawn	4.94	3.96		1.15	0.80-1.67	1.17	0.82-1.67	1.18	0.81-1.73
	Intensity of pain alleviation	27.46	28.31		1.11	0.88-1.40	0.97	0.77-1.22	1.13	0.88-1.47
	Medication to hasten death	0.33	0.96		0.44	0.15-1.28	0.35	0.12-0.99	0.43	0.15-1.27
	None of the above	24.00	22.95		1.44	1.08-1.71	1.09	0.87-1.36	1.39	1.09-1.78
**Patient gender**				.134						
	Men	52.43	50.01				1		1	
	Women	47.57	49.99				1.00	0.87-1.15	1.03	0.89-1.19
**Patient age**				.001						
	18-49	5.43	5.06				1		1	
	50-69	21.93	17.43				1.21	0.86-1.15	1.23	0.87-1.75
	70-79	21.86	19.24				1.08	0.77-1.52	1.19	0.84-1.70
	80-89	37.00	39.22				0.86	0.62-1.19	1.06	0.75-1.50
	90+	13.78	19.05				0.67	0.46-0.96	0.90	0.61-1.33
**Place of death**				.001						
	Hospital	64.10	56.52				1		1	
	Home	21.07	24.00				0.76	0.64-0.92	1.04	0.81-1.36
	Nursing/retirement home	12.03	16.69				0.73	0.58-0.90	1.02	0.78-1.32
	Other	2.80	2.79				0.87	0.56-1.32	1.20	0.75-1.91
**Cause of patient death**				.033						
	Cancer	25.19	28.45	.019			1		1	
	Cardiovascular disease	24.49	24.32	.900			1.41	1.14-1.74	1.11	0.88-1.39
	Neurological disease	13.91	14.90	.362			1.31	1.04-1.65	1.08	0.84-1.38
	Infectious disease	8.80	6.28	.003			1.78	1.35-2.34	1.32	0.98-1.77
	Respiratory disease	6.76	6.52	.729			1.39	1.04-1.86	1.12	0.82-1.53
	Digestive disease	5.00	4.03	.113			1.42	1.00-2.00	1.18	0.82-1.71
	Mental health	3.01	3.12	.819			1.46	0.96-2.22	1.35	0.87-2.09
	Violent death	3.07	3.59	.417			1.03	0.67-1.58	0.80	0.51-1.27
	Other	9.77	8.78	.300			1.56	1.19-2.06	1.35	0.87-2.09

Controlling for patient characteristics, Model 2 shows that deaths of people aged 90 and over were less often reported using the Web-based questionnaire, as were deaths outside the hospital. Furthermore, compared to cancer, most other causes of death (except violent or sudden death) were reported more often in the Web-based questionnaire. The administration of medication to hasten death was reported less in the Web-based questionnaire (OR 0.35, 95% CI 0.12-0.99).

When both physician and patient characteristics were controlled (Model 3), only physician characteristics were significantly associated with response mode. Regarding end-of-life decisions, reporting “no decision” was more prevalent for the Web-based questionnaire (OR 1.39, 95% CI 1.09-1.78), as in Model 1.

In all three models, medication to hasten death tended to be reported less through the Web-based questionnaire (although, significantly, only in two models). Thus, choosing the Web-based questionnaire was associated mainly with physician characteristics, but Web-based questionnaires still presented specificities compared to paper questionnaires: reports of medications to hasten death were rarer, whereas those of “no medical decision” were more frequent.

## Discussion

### Principal Findings

This is the first mixed-mode survey on end-of-life medical decisions. It followed most of the methodological recommendations in the literature for improving response rates [17-19]. Phone calls and postal reminders had a strong impact on participation (Figure 1), and all of these efforts contributed to a robust sample of respondents, despite a modest response rate (40.02%).

Female physicians were more likely to respond than males, as were physicians working in hospitals compared to those in private practice, while paper questionnaires were favored over Web-based questionnaires. Nevertheless, Web-based questionnaires were favored by physicians with certain characteristics: males, specialists, those who were younger, those working in neurology, emergency, and geriatrics, and those in large towns and institutions. Reports of medication to hasten death were also rarer for Web-based questionnaires (0.33% vs 0.96%), while the multivariate results suggested that there might have been fewer reports of illegal decisions through Web-based questionnaires, even when adjusting for physician characteristics.

The following paragraphs focus on the interpretation of these results and provide comments on the efficacy and drawbacks of our survey protocol.

#### Mixed-Mode Methodology

As our mixed-mode protocol used only self-administered questionnaires, the effect of the data collection mode on results may have been limited. In the case in which the selection of the data collection mode is controlled, data quality may be slightly higher by Internet [33] due to higher internal consistency. We wondered if this was the case and concluded first that the paper questionnaire was favored over the Web-based questionnaire, as found in previous studies in Australia, the United States, and Canada [21,22,34]. Secondly, the fact that some types of physicians favored the Web-based questionnaire was in line with the literature about mixed-mode surveys in the general population (young males living in large cities are more likely to respond by Internet); however, others were specific to the working conditions of the physicians (working in an institution instead of private practice), while others merit further investigation, such as medical specialty (neurology and emergency). Multivariate regressions showed that answering on the Internet was mainly associated with physicians’ characteristics, but Web-based questionnaires still presented some specificities: medication to hasten death was more rarely reported and the absence of a medical decision was more frequently reported. Thus, Web-based responses seem to concern less problematic (controversial or illegal) decisions. The choice of the Internet may reflect a selection effect related to the medical practice and typical type of medical decision made by the physician or a deliberate choice of the Internet for less problematic deaths. On the other hand, it may be a true data collection mode effect; for example, a physician who responds on the Internet may be more reluctant to declare controversial or illegal decisions, regardless of the type of death or medical decision. With the absence of randomization and nonresponse, disentangling the two is impossible. Because of the specificity of the respondents and the topics covered in the most sensitive questions, we cannot determine the direction and magnitude of the bias that occurred for each data collection.

We also noticed that the effects of the follow-up interventions (phone calls and postal reminders) were lower for Web-based questionnaires than for postal questionnaires. This is also in line with the literature on Web-based surveys, which shows that if people do not participate immediately, they tend to feel less concerned about a survey within a short time after they have been contacted. It may also be due to the absence of any email reminders.

According to Scott et al [35], using a simultaneous mixed mode may not be the most efficient protocol for surveying physicians: it is better than using only a Web-based survey, but it is costlier. Furthermore, it provides no further benefits than using a sequential mixed-mode that begins on the Internet. Unfortunately, the opposite sequence was not compared. It is likely that the mixed-mode increased the participation rate compared to either a Web-based or paper only survey, but we cannot measure to what extent. In our protocol, the reduction in costs was not substantial compared to a pure paper survey, because all contacts were made by post and only a small number chose to answer by the Internet.

#### Identification of the Deceased Person

We found that 311 questionnaires were related to a different month of death than the one expected. The reasons for this may be: (1) physicians reported on the month they participated in the survey rather than the month the person died; (2) they did not understand or see that the questions were about a specific case; (3) they did not have access to the person’s file but still wanted to participate, so they chose another case; (4) they wanted to respond about a specific case that—from their point of view—was more interesting. The significant differences between retained and discarded questionnaires suggest that, in some cases, these physicians may indeed have purposely chosen what they believed to be a “more interesting case.”

### Comparison With Other Studies

The methodology of our survey was similar to the one applied by earlier nationwide epidemiological postal surveys in Europe [4,25]; however, it used a mixed-mode strategy with telephone call backs and postal reminders from different medical authorities. The trusted party was not allowed or able to link the characteristics of the deceased to the questionnaire sent by the physician, but we showed that asking for some characteristics of the deceased person allowed us to check for possible errors in their identity, which contrasts with previous Belgian surveys [8,36]. We did not use a lawyer as a third party: a preliminary discussion in France concluded that this choice would cause physicians to be reluctant and thus undermine the confidentiality and anonymity that we sought. The use of a specialized hospital department was well received (did not provoke any comment).

Our response rate was modest but comparable to what has been commonly found in other surveys among physicians in France [37]. Furthermore, we attained almost twice the response rate that was recently reported in a representative survey of all practitioners in Australia [35]. However, ours was lower than those of previous surveys of the same topic in other countries [4,8,36]. One reason for this low rate may be the fact that a large proportion of the physicians felt unconcerned, because they thought the deaths they were in charge of were rather ordinary (sudden death or death without any particular end-of-life decision).

### Limitations and Recommendations for Future Surveys (in France and in Other Countries)

An apparent weakness is that we did not provide any incentive to improve our response rate. Prior research has demonstrated that prepaid monetary incentives (rather than nonmonetary) were effective in promoting survey participation [38-40]. In France, monetary incentives for surveys conducted by public institutions are uncommon, as opposed to studies sponsored by the pharmaceutical industry. As a consequence, no methodological evaluation study has been published on this topic. However, the success of financial incentive is not guaranteed: in a recent national telephone survey of GPs carried out by the National Institute of Health Education and Prevention in France, many participants refused the €30 incentive (equivalent to 1.5 times the consultation fee) to participate [41], arguing that this proved the survey may have had a commercial purpose. In the context of the EOLF survey, it might have been perceived as inappropriate. Nonmonetary incentives [39,40] may also improve participation, but it is not guaranteed, as demonstrated by a randomized, controlled experiment conducted among physicians in France [37]. In EOLF, 3.03% of refusals were explicitly linked to the absence of financial incentive; even if underreported, the effect on the participation rate was most likely marginal.

The fact that 19% of the contacted physicians in the call-back phase and 4.81% of the physicians in the nonrespondent survey were not aware of the original survey suggests that a phone call is necessary to overcome postal mailing problems, and we strongly recommend it for future surveys. For the same reason, although we could not assess the performance of this procedure, we recommend implementing public campaigns and reminders by medical authorities.

For data protection purposes, we made sure that no linkage was possible. This has three important consequences. (1) Due to the anonymity process, we were unable to eliminate potential paper and Internet questionnaires related to the same deceased person (if any). Some of the physicians used the same logins and passwords for all of their questionnaires and therefore generated tedious work for reconstituting each case. (2) The weighting process of the survey had to be simple, as no detailed information merging the initial sample and the respondent sample could be used, except when using aggregated data. (3) It is impossible to compute an accurate response rate by physician characteristics, because we could not merge the information in the sampling frame to that in the contact files and questionnaires. Future surveys must take these constraints into account. Our results nevertheless show that a good option for ensuring consistency in the questionnaire is to ask for broad characteristics of the deceased instead of linking them from the death certificate.

With a clear preference for the paper questionnaire and differences in reporting some important medical decisions, it seems too early to consider only an Internet-based survey in France. As precluding the internet does not appear to be an option currently—especially among young physicians—and as the topic and respondents of surveys on end-of-life medical decisions are very specific, we recommend conducting a mixed mode survey. Nevertheless, future research is needed for defining the best protocol (simultaneous vs sequential paper and Web-based), as well as for controlling selection and measurement effects in the data collection mode.

## Abbreviations

AAPOR: American Association for Public Opinion Research
CCTIRS: Comité Consultatif sur le Traitement de l’Information en matière de Recherche dans le domaine de la Santé
EOLF: End of Life in France
GP: general practitioners
INED: Institut National d’Études Démographiques
INSERM: de l’Institut National de la Santé et de la Recherche Médicale
OR: odds ratio
